# Pain on the stage: a historical-neurological analysis of Afife Jale, Türkiye's first Muslim actress

**DOI:** 10.1055/s-0046-1829029

**Published:** 2026-10-06

**Authors:** Devrimsel Harika Ertem, Yavuz Altunkaynak

**Affiliations:** 1 Silivri Anadolu Hospital, Department of Pain Management, Istanbul, Türkiye.; 2 Istanbul Cam Sakura City Hospital, Department of Neurology, Istanbul, Türkiye.

**Keywords:** Analgesics, Opioid, Hyperalgesia, Drug-Related Side Effects and Adverse Reactions, History of Medicine

## Abstract

Afife Jale (1902–1941) was a revolutionary in Turkish history as the first Muslim woman to perform on the professional theater stage. Despite her cultural significance, her life was overshadowed by chronic, debilitating headaches, and a subsequent fatal struggle with substance use disorder. This historical reconstruction explores the intersection of her pioneer identity, the progression of her neurological condition, and the iatrogenic medical practices of the early 20th century. Historical accounts suggest that Jale may have experienced recurrent severe hemicranial pain, exacerbated by the sociopolitical stress of her era. Her exposure to morphine as a primary analgesic, a common practice of the period, may have contributed to a clinical evolution compatible with phenomena currently conceptualized as medication-overuse headache and opioid-induced hyperalgesia. Her decline and eventual death at age 39 illustrate the tragic consequences of failing to distinguish between physical pain and psychological trauma within the therapeutic paradigms of the early Republican era. Her story is a reminder that, despite new treatments for headache, iatrogenic addiction remains a serious risk. Honoring her legacy means committing to ethical, multidisciplinary pain management that protects both the health and the creative soul of every patient.

## INTRODUCTION

The relationship between chronic primary headaches, specifically migraine, and artistic creativity has long been a subject of clinical interest. 1 2 3 4 Historical and epidemiological data suggest a high prevalence of migraine among renowned artists, often influencing their aesthetic perceptions and creative outputs. 3 Migraine aura and the sensory hypersensitivity associated with these cephalalgias have frequently been cited as both a burden and a source of distorted visual or emotional inspiration. 2 4 However, beyond the creative spark, the management of these debilitating pains has historically been a double-edged sword, where the quest for relief often led to catastrophic clinical outcomes before the advent of modern neuropharmacology.

Afife Jale (1902–1941) was born in Istanbul during the final years of the Ottoman Empire and became the first Muslim Turkish woman to perform professionally on the theater stage. 5 At a time when Muslim women were prohibited from acting in public performances, her appearance on stage represented a remarkable challenge to prevailing social and cultural norms. 6 Beyond her artistic achievements, she emerged as an important symbol of women's emancipation and modernization during the transition from the Ottoman Empire to the Republic of Türkiye. Her pioneering role has secured her a lasting place in Turkish cultural history ( Figure 1 ).

**Figure 1:**
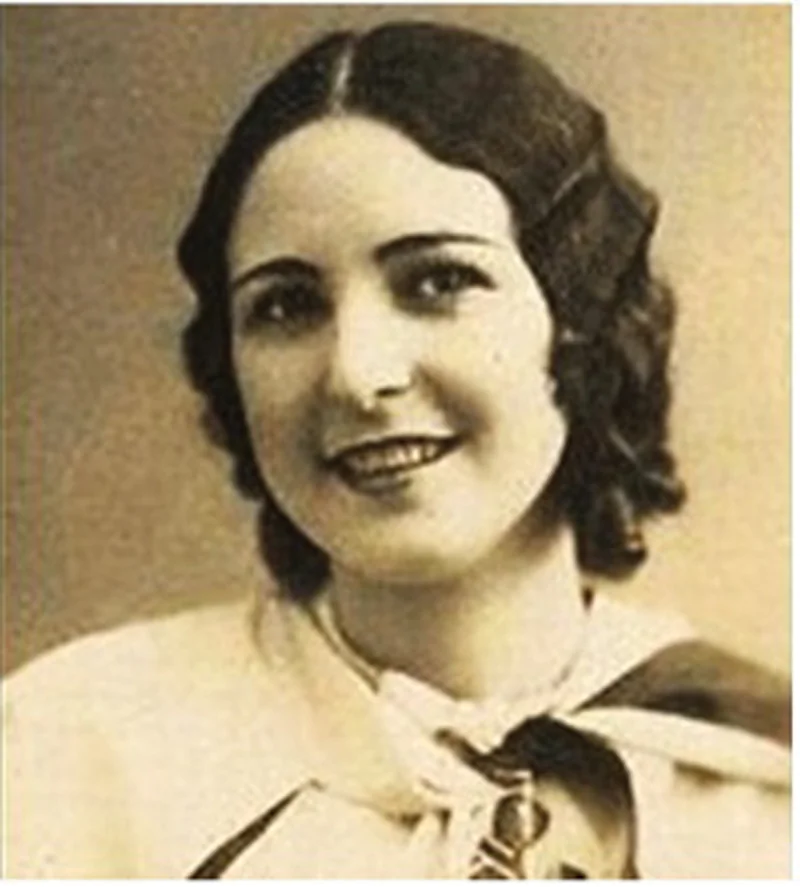
Afife Jale (1902–1941).

Behind her public defiance lay a private struggle with excruciating, refractory headaches, which many historians and contemporary medical scholars believe to be severe possible chronic migraine. The tragic trajectory of her life was irrevocably altered when she was introduced to morphine as a primary analgesic, a common but perilous medical practice of the early 20th century. 6 7 This iatrogenic opioid dependence, exacerbated by the psychological trauma of her professional exile, eventually led to her institutionalization at the Bakırköy Psychiatric Hospital and her untimely death. 7 This article reexamines Jale's medical history through the lens of modern pain medicine to honor her legacy and analyze the evolution of headache management.

## THE CHRONICLE OF PAIN: SOCIOPOLITICAL STRESS AND CEPHALALGIA

In the early 1920s, Afife Jale began experiencing recurring episodes of severe, throbbing hemicranial pain accompanied by photo- and phonophobia. These symptoms may be considered compatible with contemporary descriptions of chronic migraine without aura. Due to the lack of accessible primary medical records regarding Jale's family, it remains impossible to ascertain whether there was a hereditary predisposition or a familial history of primary headache disorders.

Historical reconstruction suggests that these paroxysms were severely exacerbated by the profound emotional distress of her professional exile and the constant threat of legal persecution. In this era, the boundaries between physical nociception and psychological trauma were frequently blurred, leading to a state of central sensitization where emotional agony and physical pain became indistinguishable.

## THERAPEUTIC PARADIGMS OF THE ERA: THE “DR. SUAT” PERIOD AND IATROGENIC HARM

Seeking relief for her refractory headaches, Jale was attended by a local physician, identified in historical records as Dr. Suat. 7 In the absence of a structured neurological assessment or a long-term management plan, the physician initiated a treatment regimen consisting of subcutaneous morphine injections. 8 At the time, the rapid analgesic effect of opioids was mistakenly prioritized over their high addictive potential.

While Jale initially reported transient relief, the intervention led to rapid opioid tolerance. This period represents a critical failure in medical ethics and practice, as the frequency of injections transitioned her condition from an episodic neurological disorder to a complex state of iatrogenic dependence.

## CLINICAL DECLINE AND THE MEDICAL OVERUSE SPIRAL

By the late 1920s, Jale's condition had progressed into daily, chronic headaches. From a contemporary neurological perspective, this pattern could be interpreted as being compatible with medication-overuse headache (MOH) and opioid-induced hyperalgesia (OIH), where the treatment paradoxically worsened the original pain. 9 10 11

Nevertheless, the absence of detailed treatment records, dosage information, and longitudinal clinical observations precludes definitive conclusions regarding these mechanisms. Her marriage to composer Selahattin Pinar (1902–1960) in 1929 was marked by this escalating addiction. Despite the isolation of ergotamine tartrate in 1918, targeted vascular stabilizers were not yet part of the standard therapeutic toolkit in her region. Consequently, her health deteriorated into severe malnutrition and cachexia, leading to her withdrawal from the theater, the very essence of her identity.

## INSTITUTIONALIZATION AND FINAL YEARS AT BAKIRKÖY PSYCHIATRIC HOSPITAL

The final stage of her clinical journey began with her admission to the Bakırköy Psychiatric Hospital, short for Bakırköy Mazhar Osman Mental Health and Neurological Diseases Education and Research Hospital (Turkish: Bakırköy Prof. Dr. Mazhar Osman Ruh Sağlığı ve Sinir Hastalıkları Eğitim Araştırma Hastanesi), which was then directed by the pioneer Dr. Mazhar Osman (1884–1951). Historical accounts describe features suggestive of depressive symptomatology, such as signs of advanced substance use disorder, severe depressive symptoms, and social isolation. She was reportedly treated in the “indigent ward,” reflecting the tragic social fall of a national pioneer. On July 24, 1941, at the age of 39, she succumbed to a multiorgan failure secondary to chronic toxicity and malnutrition, likely a result of chronic substance toxicity and end-stage physical exhaustion.

The clinical timeline of Afife Jale's headache progression and the subsequent development of opioid dependence is summarized in Table 1 .

**Table 1:** Historical timeline of Afife Jale's reported symptoms and contemporary neurological interpretation

Period	Historical events and reported clinical features	Contemporary neurological interpretation*
Early 1920s	Reported episodes of severe unilateral throbbing headache associated with sensitivity to light and sound.	Symptoms may be considered compatible with episodic migraine according to contemporary headache classifications.
1920–1923	Professional debut accompanied by intense socio-political pressure, police raids, and social exclusion.	Chronic psychosocial stress may have contributed to pain amplification processes resembling contemporary concepts of central sensitization.
Mid 1920s	Reported initiation of morphine injections for headache relief.	Early exposure to opioid-based analgesia with potential risk of tolerance and dependence.
Late 1920s	Historical accounts describe increasing headache frequency and escalating morphine use.	Clinical evolution may have been compatible with phenomena currently conceptualized as MOH and/or OIH.
1929–1930s	Marriage to Selahattin Pınar and progressive physical decline described in biographical sources.	Possible opioid dependence accompanied by deterioration in general health status.
1930s–1941	Admission to the Bakırköy Psychiatric Hospital and reports of social isolation during her final years.	Historical descriptions suggest psychological distress and chronic substance exposure; definitive psychiatric or neurological diagnoses cannot be established retrospectively.
July 24, 1941	Death at age 39.	Exact cause of death remains uncertain; historical accounts describe severe physical deterioration during the final stage of life.

Abbreviations: MOH, medication-overuse headache; OIH, opioid-induced hyperalgesia.

Notes: All neurological interpretations presented in this table are retrospective and hypothetical. They are based on historical and biographical accounts rather than contemporaneous clinical examinations, diagnostic records, or treatment documentation.

## INTERPRETATIVE ANALYSIS: MIGRAINE, MORPHINE, AND THE MEDICAL ERA

### **The phenomenon of central sensitization and emotional pain**

In the case of Afife Jale, the boundaries between physical nociception and psychological trauma were significantly blurred. From a pain medicine standpoint, her chronic headaches likely represented a state of Central Sensitization. Central sensitization is believed to underlie many of the temporal and spatial alterations in pain processing, along with reductions in pain thresholds, observed in acute and chronic clinical pain, thereby emphasizing the central nervous system's key role in the emergence of pain hypersensitivity. 12 The constant socio-political stress, combined with the “fight or flight” response triggered by police raids, may have led to a dysfunctional descending inhibitory pain pathway. It is conceivable that prolonged psychosocial stress may have contributed to pain amplification processes that resemble what is currently described as central sensitization, although such a mechanism cannot be directly demonstrated retrospectively.

### **Iatrogenic complications: opioid-induced hyperalgesia (OIH)**

The intervention by Dr. Suat using morphine injections is a classic, albeit tragic, example of iatrogenic harm. Modern neurology recognizes Opioid-Induced Hyperalgesia (OIH), where long-term opioid use paradoxically lowers the pain threshold and worsens the original headache. 13 It is highly probable that Afife Jale's escalating pain was not a failure of the drug, but a direct neurochemical consequence of it. Furthermore, the clinical picture likely evolved into MOH, creating a feedback loop of pain and dependence that the medical knowledge of the 1930s was ill-equipped to break. 14

From a contemporary perspective, Jale's clinical course highlights the limitations of headache management strategies available during the early 20 ^th^ century. At that time, physicians had limited understanding of opioid dependence, medication-overuse headache, and the long-term neurobiological consequences of chronic opioid exposure. Morphine was widely regarded as an effective analgesic, and the concepts of addiction to medicine, structured risk assessment, and formal informed consent had not yet evolved into their modern forms. Rather than reflecting individual clinical negligence, Jale's case may be viewed as an illustration of the therapeutic constraints and medical knowledge of the period. Her experience underscores how advances in neurology, pain medicine, and medical ethics have transformed contemporary approaches to chronic headache management.

### **Differential diagnostic considerations**

Although the historical descriptions of Afife Jale's symptoms may be considered compatible with migraine, alternative explanations should also be acknowledged. The available historical accounts are limited and do not permit definitive retrospective diagnosis. Recurrent severe headaches could potentially have reflected other primary headache disorders, including chronic daily headache syndromes.

Furthermore, medication toxicity related to prolonged morphine exposure may itself have contributed to the progression and persistence of pain symptoms. Functional neurological symptoms, psychological distress, and somatic manifestations of chronic psychosocial stress may also have influenced the clinical picture.

Finally, the absence of contemporaneous medical evaluations precludes exclusion of systemic or neurological conditions that might present with chronic headache. Consequently, migraine should be regarded as a plausible rather than definitive explanation for Jale's reported symptoms.

### **The lost opportunities in early 20th century therapeutics**

While ergotamine tartrate was isolated in 1918 by Arthur Stoll (1887–1971), 15 its widespread clinical use for migraines was still in its infancy during Jale's most desperate years. Had she been managed with targeted vascular stabilizers rather than Mu-opioid receptor agonists, her cognitive functions and artistic career might have been preserved. Historical accounts indicate that Jale spent part of her final years at Bakırköy Psychiatric Hospital. Secondary biographical sources describe progressive physical decline, social isolation, and longstanding morphine use, although the absence of accessible clinical records precludes definitive assessment of her psychiatric and neurological status.

## METHODOLOGICAL CONSIDERATIONS AND LIMITATIONS OF RETROSPECTIVE DIAGNOSIS

The present analysis should be interpreted within the limitations inherent to retrospective neurohistorical investigation. No primary neurological examinations, diagnostic records, neuroimaging studies, or detailed treatment documents concerning Afife Jale are currently available. Consequently, any proposed neurological diagnosis remains hypothetical and should not be regarded as definitive. The clinical interpretations presented in this article are based on historical descriptions recorded in biographical sources and secondary accounts, which may be affected by reporting bias, selective recollection, and incomplete documentation.

Furthermore, contemporary diagnostic frameworks such as chronic migraine, medication-overuse headache, opioid-induced hyperalgesia, central sensitization, and opioid use disorder were developed many decades after the events described and may not fully capture the historical and sociocultural context in which these symptoms occurred. Therefore, the diagnoses discussed in this manuscript should be viewed as clinical interpretations intended to facilitate interdisciplinary discussion rather than as established historical facts.

## AN ACT OF HISTORICAL RESTITUTION

The life of Afife Jale serves as a poignant reminder that the management of chronic pain is not merely a technical task, but a fundamental commitment to preserving human dignity. Her journey illustrates the tragic consequences that arise when medical practice fails to distinguish between the biological roots of pain and the heavy toll of psychological trauma.

In the early 20th century, the reliance on potent but risky sedatives for migraines, without understanding their long-term impact on the brain's pain processing, turned a manageable condition into a fatal cycle of dependency. From a modern perspective, her decline was not a personal failure, but the result of a medical era that lacked the tools to break the feedback loop of pain and addiction. As clinicians with experience in headache medicine, we believe that reexamining Jale's case offers a valuable opportunity to explore the historical evolution of headache diagnosis, pain management, and opioid prescribing practices. Although the medical world has moved beyond the dangerous practices of her time, the risk of mismanaging chronic pain remains a global concern.

Afife Jale's case highlights the importance of multidisciplinary approaches to chronic pain management and the need to consider both biological and psychosocial contributors to suffering. Although modern headache medicine has advanced substantially since Jale's lifetime, her case remains relevant as a historical example of the challenges associated with chronic pain treatment and the unintended consequences of therapeutic interventions.
